# Loss of Anticodon Wobble Uridine Modifications Affects tRNA^Lys^ Function and Protein Levels in *Saccharomyces cerevisiae*

**DOI:** 10.1371/journal.pone.0119261

**Published:** 2015-03-06

**Authors:** Roland Klassen, Pia Grunewald, Kathrin L. Thüring, Christian Eichler, Mark Helm, Raffael Schaffrath

**Affiliations:** 1 Institut für Biologie, Fachgebiet Mikrobiologie, Universität Kassel, Kassel, Germany; 2 Institut für Pharmazie und Biochemie, Johannes Gutenberg Universität Mainz, Mainz, Germany; University of Florida, UNITED STATES

## Abstract

In eukaryotes, wobble uridines in the anticodons of tRNA^Lys^_UUU_, tRNA^Glu^_UUC_ and tRNA^Gln^_UUG_ are modified to 5-methoxy-carbonyl-methyl-2-thio-uridine (mcm^5^s^2^U). While mutations in subunits of the Elongator complex (Elp1-Elp6), which disable mcm^5^ side chain formation, or removal of components of the thiolation pathway (Ncs2/Ncs6, Urm1, Uba4) are individually tolerated, the combination of both modification defects has been reported to have lethal effects on *Saccharomyces cerevisiae*. Contrary to such absolute requirement of mcm^5^s^2^U for viability, we demonstrate here that in the *S*. *cerevisiae* S288C-derived background, both pathways can be simultaneously inactivated, resulting in combined loss of tRNA anticodon modifications (mcm^5^U and s^2^U) without a lethal effect. However, an *elp3* disruption strain displays synthetic sick interaction and synergistic temperature sensitivity when combined with either *uba4* or *urm1* mutations, suggesting major translational defects in the absence of mcm^5^s^2^U modifications. Consistent with this notion, we find cellular protein levels drastically decreased in an *elp3uba4* double mutant and show that this effect as well as growth phenotypes can be partially rescued by excess of tRNA^Lys^_UUU_. These results may indicate a global translational or protein homeostasis defect in cells simultaneously lacking mcm^5^ and s^2^ wobble uridine modification that could account for growth impairment and mainly originates from tRNA^Lys^_UUU_ hypomodification and malfunction.

## Introduction

Transfer RNA (tRNA) is known to undergo extensive modification of nucleobases, including uridines at position 34, which represents the first base of the anticodon that can engage in non-canonical base pairing (wobbling) with the third base of the codon (reviewed in [1]). In the yeast *Saccharomyces cerevisiae*, wobble uridines (U34) in the anticodons from 11 tRNA species were shown to undergo modification to either 5-methoxy-carbonyl-methyl-uridine (mcm^5^), 5-carbamoyl-methyl-uridine (ncm^5^), carbamoylmethyl-2′-O-methyluridine (ncm^5^Um) or 5-methoxy-carbonyl-methyl-2-thio-uridine (mcm^5^s^2^U) [2, 3]. Synthesis of the side chains mcm^5^ and ncm^5^ requires the Elongator complex (Elp1-Elp6) as well as factors involved in interaction with and modification of Elongator (Kti11-Kti13; Sit4, Sap180/Sap195; Hrr25/Kti14) [2, 4–13]. Formation of mcm^5^, but not ncm^5^ further requires a methyltransferase complex (Trm9/Trm112) [14–16] and removal of the latter results in the replacement of mcm^5^ side chains on wobble uridines with ncm^5^ [16]. Wobble uridine thiolation, which is required for completed formation of the mcm^5^s^2^ modification in tRNA^Lys^_UUU_, tRNA^Gln^_UUG_ and tRNA^Glu^_UUC_, appears to occur independently of mcm^5^ formation on the same base. Consistent with this, Elongator mutants were shown to accumulate s^2^U [2]. Similarly, mutants lacking components of the sulfur transfer system required for U34 thiolation (Urm1, Uba4, Ncs2, Ncs6) no longer form mcm^5^s^2^U but instead show elevated levels of mcm^5^U, suggesting that the pathways required for modifying U34 at position 2 and 5 operate, at least in part, independently of each other [17, 10, 16]. Elongator mutants or thiolation-minus cells have been shown to exhibit pleiotropic phenotypes, including, but not restricted to, elevated resistance to fungal anticodon nuclease toxins, cell cycle delay, slow growth at 30°C, thermosensitivity and sensitivity to various exogenous stresses [18–26]. In addition to its role in sulfur transfer to tRNA, Urm1 itself can act as a ubiquitin like protein and becomes covalently conjugated to target proteins, a process termed urmylation [22]. It was demonstrated that the combination of *elp3* and *ncs6* mutations (resulting in the simultaneous loss of both, mcm^5^ chain formation and s^2^ thiolation) causes synthetic lethality and inviability in *S*. *cerevisiae* [17]. Importantly, inviability could be suppressed by overexpression of tRNA^Lys^_UUU_, which normally carries mcm^5^s^2^U, but completely lacks U34 modifications in the background of the *elp3ncs6* double mutant [17]. Therefore, inviability was interpreted to result from a specific decoding deficiency of tRNA^Lys^_UUU_ that is caused by the lack of wobble uridine modification and can be countered and partly compensated for by excess of the hypomodified tRNA^Lys^_UUU_ [17].

Further genetic experiments revealed that mcm^5^ and mcm^5^s^2^ modifications in general can be regarded as factors improving the decoding efficiency of tRNAs in ways that involve canonical and non-canonical anticodon-codon base pairing [3]. In line with a decoding defect underlying phenotypes of mcm^5^/ncm^5^U or s^2^U modification mutants, not only are synthetic genetic interactions between Elongator minus and thiolation minus mutations suppressible by tRNA overexpression, but also single mutant growth phenotypes can be rescued by excess of tRNA^Lys^_UUU_ and tRNA^Gln^_UUG_ [24, 26]. Individual loss of mcm^5^/ncm^5^ modification or thiolation pathways may affect tRNA decoding and translational efficiency in a codon dependent fashion, since the mcm^5^s^2^ modified tRNAs (tRNA^Lys^_UUU_, tRNA^Gln^_UUG_ and tRNA^Glu^_UUC_) are thought to be mainly involved in decoding of the A-ending codons for lysine (AAA), glutamine (CAA) and glutamic acid (GAA) but not the G-ending ones [3]. Consistently, a genome wide study in *S*. *cerevisiae* recently confirmed major negative effects of *elp3* and *urm1* single mutations on the translatability of transcripts enriched for A-ending codons [27]. However, an independent study by Zinshteyn and Gilbert [28] claimed these effects were too minor to affect global protein outputs, though a slowdown of translation at AAA, CAA, and GAA codons in the absence of mcm^5^/ncm^5^ or s^2^U was also confirmed by the authors. These studies on the role of mcm^5^s^2^U in *S*. *cerevisiae*, however, are hampered by the thought that complete removal of mcm^5^s^2^U is not tolerated and results in inviability, hence the effects on translation and phenotypes in the full absence of mcm^5^s^2^U could not be studied yet.

We show here, that in the reference strain isogenic to *S*. *cerevisiae* S288C, complete removal of the U34 wobble modification mcm^5^s^2^ is tolerated at the expense of a growth defect. Further, the strain lacking mcm^5^s^2^U yields reduced total protein levels, pointing to a translational defect which likely accounts for the growth-related phenotypes, for both can be suppressed by excess levels of hypomodified tRNA. Since overexpression of tRNA^Lys^_UUU_ alone has significant suppressor effects, malfunction of this tRNA is mainly responsible for phenotypes induced by complete loss of mcm^5^s^2^U.

## Results

### Simultaneous removal of wobble uridine mcm^5^ modification and thiolation

**Fig 1:**
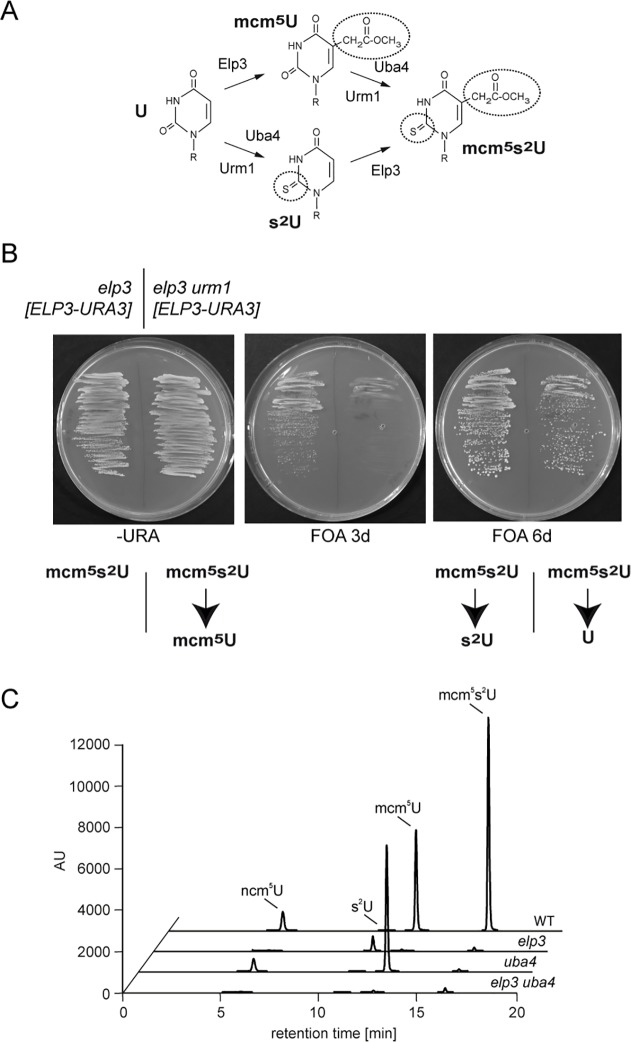
Simultaneous loss of wobble uridine mcm^5^ modification and thiolation. (A) Simplified scheme displaying the partially independent action of the wobble uridine mcm^5^ modification-(exemplified by Elp3) and thiolation pathway (exemplified by Urm1/Uba4) on the same substrate (for review see [30]). (B) Generation of *elp3urm1* double mutants by plasmid shuffling. Left section of plates: Strains carrying a genomic *elp3* deletion complemented by a *URA3* plasmid carrying wild type *ELP3*. Right section of plates: Strain carrying genomic *elp3* and *urm1* deletions and the complementing *ELP3* wild type plasmid. Both strains were streaked in parallel on –URA and FOA. Photographs were taken after 3d (-URA, FOA) and 6d (FOA) of incubation at 30°C. Below the plates, the effects on mcm^5^s^2^ wobble uridine modification status are indicated. Similar results were obtained for a likewise constructed *elp3uba4* double mutant. (C) LC-MS/MS chromatograms of ncm^5^U, s^2^U, mcm^5^U and mcm^5^s^2^U in tRNA from wild type and mutant cells. Peak heights of each sample were normalized to the injected RNA amount using the UV peaks of uridine to ensure intersample comparability of the peaks. AU – arbitrary units.

To test the effect of simultaneous removal of wobble uridine mcm^5^/ncm^5^ side chain formation and thiolation (Fig. 1A) in an *S*. *cerevisiae* S288C-derived background, we first complemented a genomic *elp3* deletion with the wild type *ELP3* gene on a centromeric *URA3* plasmid. Next, *URM1* was deleted and the resulting strain (*elp3 urm1* [*ELP3-URA3*]) grown along with its parent (*elp3* [*ELP3-URA3*]) on SC-URA and on FOA+URA. Both strains grow equally well on SC-URA, a medium used to maintain the *ELP3* wild type allele but show very distinct growth phenotypes on FOA+URA, a medium on which the *ELP3* carrying plasmid cannot be propagated due to counter-selection. Under these conditions, growth of the *elp3urm1* double mutant is severely delayed, but loss of the *ELP3* carrying plasmid is tolerated (Fig. 1B). Using this strategy, we also obtained a viable *elp3uba4* double mutant, indicating the complete absence of mcm^5^s^2^ can be tolerated and may not necessarily cause synthetic lethality in *S*. *cerevisiae*. This is in stark contrast to a previous report [17] on the inviability of another yeast strain (W303 background) lacking mcm^5^s^2^U. It should be noted, that the W303 genome differs detectably from the S288C reference genome [29], hence differential sensitivities to the entire removal of wobble uridine modifications are not entirely unexpected.

**Fig 2:**
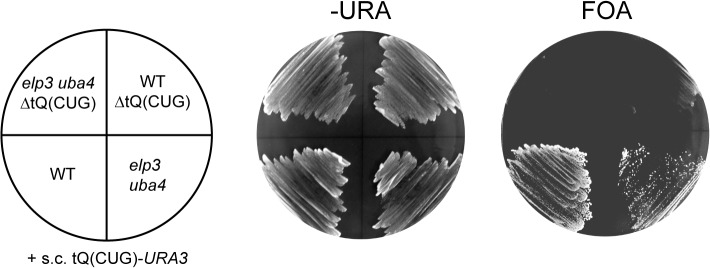
Deletion of the tRNA^Gln^_CUG_ gene (ΔtQ(CUG)) causes inviability in WT and *elp3uba4* double mutants. WT and *elp3uba4* strains were transformed with the single copy *URA3* plasmid pAK01 carrying the tRNA^Gln^_CUG_ gene and subsequently, the genomic copy of the gene was deleted. Strains were grown in parallel on –URA and FOA medium.

To verify the simultaneous absence of mcm^5^ and s^2^ modifications in *S*. *cerevisiae*, we isolated total tRNA from the *elp3uba4* double mutant, the *elp3* and *uba4* single mutants and the isogenic parental wild type strain and subjected these to LC-MS/MS analysis. Consistent with previous reports, *elp3* mutants are defective in the formation of mcm^5^U, mcm^5^s^2^U and ncm^5^U but accumulate s^2^U, which is not detected in the wild type (Fig. 1C), thus reconfirming the notion that U34 thiolation can occur in the absence of mcm^5^ formation (Fig. 1A; [2]). The *uba4* strain is proficient in the formation of mcm^5^U and ncm^5^U but specifically lacks mcm^5^s^2^U as well as s^2^U, demonstrating the wobble thiolation defect. In the *elp3uba4* double mutant and similar to *elp3* cells alone, no mcm^5^U, mcm^5^s^2^U or ncm^5^U is detectable but at the same time, formation of s^2^U is also abolished (Fig. 1C). This result indicates a defect in both wobble uridine mcm^5^/ncm^5^-modification and thiolation in the combined absence of *ELP3* and *UBA4*.

### Effect of tRNA wobble uridine modification defects on wobble decoding and cellular protein synthesis

We first checked morphology of the *elp3uba4* double mutant in comparison to the wild type (S1 Fig.). We found a small number of irregularly shaped cells containing elongated and/or multiple buds which were not observed for the isogenic wild type. There were no other significant differences between wild type and the *elp3uba4* double mutant with respect to cell size or nuclear morphology (S1 Fig.). As an initial test to the functional role of mcm^5^s^2^U, we asked whether the complete absence of the modification might affect the ability of tRNA^Gln^_UUG_, to recognize non-cognate codons. It was shown previously that mcm^5^s^2^U modified tRNA^Gln^_UUG_ is able to decode the alternative CAG codon via U/G wobbling when present at elevated levels but not under normal circumstances [3]. Thus, high copy tRNA^Gln^_UUG_ suppresses the lethal effect of a deletion in *SUP70*, the only gene for tRNA^Gln^_CUG_ [3]. Since tRNA^Leu^_UAG_, the only known yeast tRNA naturally carrying an unmodified wobble uridine is capable of efficient decoding of all four CUN codons by wobble base pairing [2, 31], it appeared possible that complete absence of wobble uridine modifications might also increase the ability of tRNA^Gln^_UUG_ to decode the CAG codon via U/G wobbling. To test this idea, we introduced a *URA3* plasmid carrying the tRNA^Gln^_CUG_ gene into WT and *elp3uba4* double mutants and subsequently deleted the single chromosomal gene for tRNA^Gln^_CUG_. On FOA medium, the *URA3* plasmid-bourne tRNA^Gln^_CUG_ cannot be maintained, resulting in complete growth arrest of WT cells due to the inability of tRNA^Gln^_UUG_ to efficiently decode the CAG codon [3]. Growth assays further revealed that the *elp3uba4* double deletion did not suppress the inviability on FOA medium (Fig. 2), indicating that tRNA^Gln^_UUG_ carrying an unmodified wobble uridine is also inable to efficiently decode the CAG codon. Hence, mcm^5^s^2^U is not responsible for the general inability of tRNA^Gln^_UUG_ to efficiently read the alternative G-ending codon.

**Fig 3:**
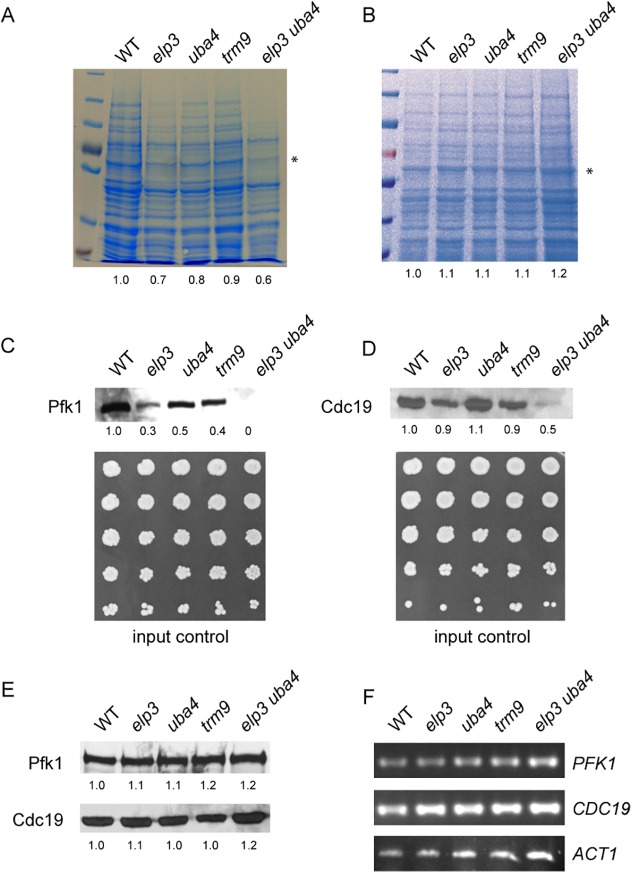
Loss of mcm^5^s^2^ affects protein levels. (A) Analysis of cellular protein content of indicated strains. Identical numbers of cells were subjected to chemical lysis and analyzed by SDS-PAGE and Coomassie staining. The band marked with (*) was quantified and relative intensities compared to WT indicated below. (B) Analysis of total protein extracts from indicated strains adjusted to identical protein concentration. The band marked with (*) was quantified and relative intensities compared to WT indicated below. (C) Western detection of Pfk1 levels from indicated strains after chemical lysis of identical numbers of cells. Signal intensities relative to WT are indicated below. Before lysis, suspensions were serially diluted and spotted on YPD to confirm equal cell densities (input control). (D) Western detection of Cdc19 levels from indicated strains after chemical lysis of identical numbers of cells. Signal intensities relative to WT are indicated below. Before lysis, suspensions were serially diluted and spotted on YPD to confirm equal cell densities (input control). (E) Western detection of Pfk1 and Cdc19 in total protein extracts from indicated strains adjusted to identical protein concentration. Signal intensities relative to WT are indicated. (F) RT-PCR analysis of cDNA from indicated strains for *PFK1*, *CDC19* and *ACT1* mRNAs. Identical amounts of total RNA were subjected to reverse transcription.

To test whether the complete absence of mcm^5^s^2^U or other incorrect wobble uridine modification scenarios may lead to protein synthesis defects, we analyzed total cellular protein levels in strains with different modification defects (Fig. 3A). We used *elp3* and *uba4* single and double mutants as well as *trm9* mutants carrying ncm^5^/ncm^5^s^2^U instead of mcm^5^/mcm^5^s^2^U [16]. For each strain, identical numbers of cells were subjected to chemical lysis and subsequently analyzed by SDS PAGE and Coomassie staining. While there were smaller effects in the *trm9*, *uba4* and *elp3* single mutants, total cellular protein content was markedly decreased in the *elp3uba4* double mutant (Fig. 3A). We also monitored differences in protein content for Cdc19 (pyruvate kinase; [32]) and Pfk1 (phosphofructokinase; [33]) and found that in general, differences in abundance are comparable to the effects on total protein levels (Fig. 3C, 3D). Both proteins are already reduced in abundance in the *elp3* single mutant. In the complete absence of mcm^5^s^2^ (*elp3uba4*), however, Cdc19 is hardly detectable, while Pfk1 becomes entirely undetectable, indicating a significant negative impact of the tRNA modification defect on the abundance of these two proteins. In comparison to *elp3* and *elp3uba4* strains, *trm9* and *uba4* single mutants less severely decreased the abundance of Pfk1. For Cdc19, levels were slightly decreased in *trm9* but not in *uba4* backgrounds (Fig. 3D). To verify equal cell numbers, portions of adjusted cell suspensions were removed before initializing chemical lysis, serially diluted and spotted on YPD. As shown in Fig. 3C and 3D, all tRNA modification mutants, including the *elp3uba4* mutant, where Pfk1 and Cdc19 signals are severely reduced, formed comparable numbers of viable cells in individual spots, indicating a similar input to chemical lysis. Since the drop dilution method might not be suitable to detect smaller changes in viable cell titers, we grew wild type cells along with the *elp3uba4* double mutant to early exponential phase (OD_600nm_ ∼0.5), adjusted both cultures to OD_600nm_ = 1 and determined exact total and viable cell titers by hemocytometry and viability plating. Indeed, there is a slight deviation (−13.9% for *elp3uba4* compared to WT) in total cell numbers which is likely attributable to above mentioned changes in morphology that affect OD_600nm_/total cell number ratios (S2 Fig.). Moreover, we find that *elp3uba4* double mutants exhibit an even greater loss of viable cell counts (−22.6% compared to WT), indicating a significant accumulation of dead cells. We confirmed this latter notion by staining dead cells with methylene blue. While these are essentially absent in the exponential WT culture, dead cells are detectable for the *elp3uba4* strain. In addition, we observed a number of spontaneously lysed cells, which stain dark in phase contrast and weakly positive with methylene blue. Interestingly, dead staining and lysis appears to correlate with the elongated/multiple bud phenotype (S2 Fig.). Thus, tRNA hypomodification-induced cell death likely contributes to the observed differences in protein levels but the extent of viability loss (22.6%) appears to be insufficient to solely explain the observed differences in protein abundance. Semi-quantitative RT-PCR analysis of total mRNA excluded the possibility of a severe drop in transcription of *CDC19*, *PFK1* or *ACT1* genes in the *elp3uba4* double mutant, strongly suggesting the significantly reduced abundance of Cdc19 and Pfk1 in this strain results from a translational rather than a transcriptional defect or a combination of a translational defect with the induction cell death (Fig. 3F).

Since tRNA modification defects might impair decoding of transcripts in a codon dependent manner, we next checked whether the observed effects on Pfk1 and Cdc19 are disproportionately high compared to the effect on global protein content. Rather than checking protein content per cell, we analyzed Cdc19 and Pfk1 abundances in protein preparations from the tRNA modification mutants after normalization to equal protein concentrations. Under such conditions, only differences in protein abundance that are disproportionate to the total protein (e.g. more strongly diminished than the average) would remain detectable. Strikingly, no differences in total protein content of such samples are detected after Coomassie staining (Fig. 3B) but also the observed differences of Pfk1 and Cdc19 abundance in the different tRNA modification mutants entirely leveled out (Fig. 3E). These results indicate that the negative impact of the various tRNA modification defects on Pfk1 and Cdc19 abundance is comparable to the proteome average and therefore is unlikely to be linked to a particular codon pattern of the respective genes and the translatability of their mRNAs.

### Suppression of negative genetic interaction between *elp3* and *uba4/urm1*

Mutations in Elongator subunit encoding genes, including *ELP3*, were previously shown to result in temperature sensitivity (TS) [19] and a single deletion in several of the genes required for tRNA thiolation (*URM1*, *UBA4*, *NCS6*) also results in a moderate TS phenotype [24, 26]. In both, mcm^5^/ncm^5^ or s^2^-deficient backgrounds (*elp3* or *ncs6* single mutants), the TS phenotypes can be complemented in part by excess of tRNA^Lys^_UUU_ and tRNA^Gln^_UUG_ [24], which suggested that the mcm^5^ and s^2^ modifications individually contribute to the decoding efficiency of these tRNAs. Since the combination of *elp3* and *ncs6* mutations was lethal in the previously studied strain background and this synthetic interaction could also be suppressed by excess of tRNA^Lys^_UUU_, it was concluded that the simultaneous absence of mcm^5^U and s^2^U from tRNA^Lys^_UUU_ results in a decoding defect so severe that it accounts for the observed inviability [17]. However, the inviability of the double mutant precluded the option to test this interpretation experimentally. Since we were able to generate a viable double mutant defective in both, mcm^5^U and s^2^U modification, we utilized this strain to test the previous conclusion directly.

**Fig 4:**
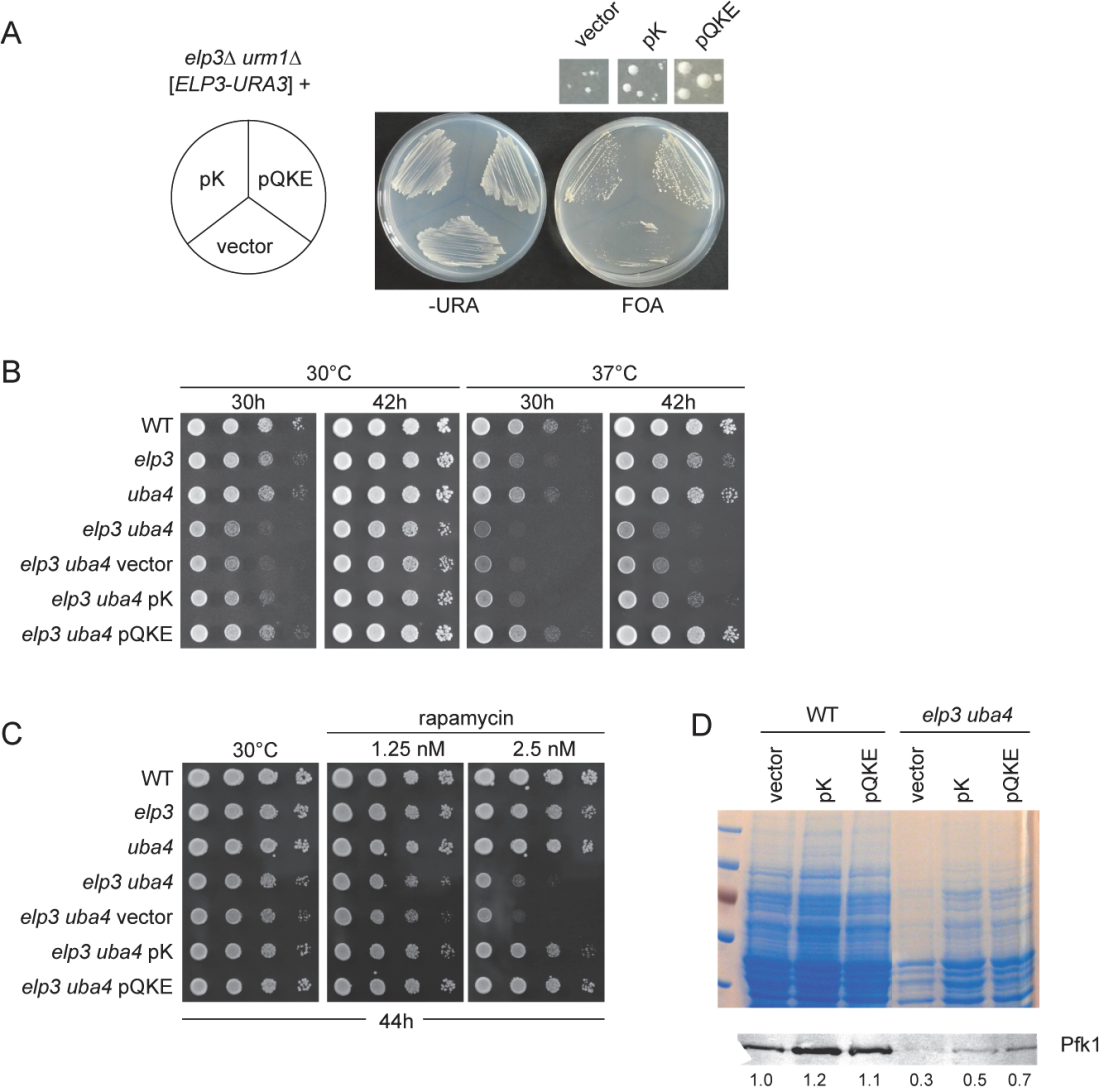
tRNA overexpression suppression of phenotypes and protein depletion induced by lack of mcm^5^s^2^U. (A) Plasmid shuffling. The *elp3urm1* strain carrying *ELP3* on a centromeric *URA3* plasmid was transformed with empty vector (pRS425) or vectors overexpressing tRNA^Lys^_UUU_ alone (pK) or together with tRNA^Gln^_UUG_ and tRNA^Glu^_UUC_ (pQKE) and subsequently streaked in parallel on –URA and FOA media. Above the FOA plate, sections corresponding to the indicated strains were magnified to illustrate colony sizes. (B) Serial dilution and spot assay with indicated strains. Replicas were incubated at 30°C or 37°C and photographed after 30h and again after 42h of incubation. (C) Serial dilution and spot assay with indicated strains on plates containing indicated amounts of rapamycin. Plates were incubated at 30°C and photographed after 44h. (D) Analysis of cellular protein content of WT and *elp3uba4* strains transformed either with empty vecor (pRS425), or vectors overexpressing tRNA^Lys^_UUU_ alone (pK) or together with tRNA^Gln^_UUG_ and tRNA^Glu^_UUC_ (pQKE). Western detection of Pfk1 levels after chemical lysis of identical numbers of cells. Signal intensities relative to WT are indicated below.

First, we analyzed a potential suppression effect of excess levels of tRNA^Lys^_UUU_ on the growth of an *elp3urm1* double mutant immediately after counterselection of the *ELP3-URA3* plasmid. The *elp3urm1* [*ELP3-URA3*] strain was transformed with plasmids overexpressing tRNA^Lys^_UUU_ alone (pK) or a combination of tRNA^Gln^_UUG_, tRNA^Lys^_UUU_ and tRNA^Glu^_UUC_ (pQKE) as well as the empty vector (pRS425). All three strains grow equally well on SC-URA (maintaining the *ELP3-URA3* plasmid) but exhibit very distinct growth phenotypes on FOA+URA, where the *ELP3-URA3* plasmid cannot be maintained due to counter-selection. On the latter medium, the empty vector control shows small colonies. In contrast to this, both pK and pQKE carrying *elp3urm1* strains exhibit significantly improved growth and colony sizes increased in the strain overexpressing the three tRNAs compared to the strain overexpressing tRNA^Lys^ alone (Fig. 4A). Thus, higher-than-normal levels of tRNA^Lys^_UUU_ lacking the wobble uridine modification have a significant suppressor effect on the slow growth of *elp3urm1* cells, and this can be further enhanced by the additional overexpression of tRNA^Gln^_UUG_ und tRNA^Glu^_UUC_.

To analyze genetic interaction of *elp3* and *urm1/uba4* with respect to TS and its suppression by excess of tRNA, we transformed the *elp3uba4* double mutant with the above mentioned tRNA overexpression plasmids and studied the resulting strains phenotypically for growth at 30°C and at 37°C along with the wild type control and the single *elp3* and *uba4* mutants. Consistent with previous reports, *elp3* and *uba4* single mutants display a TS phenotype which is more pronounced for the *elp3* strain and both mutants show a slight growth defect at 30°C (Fig. 4B). The *elp3uba4* double mutant displays strongly decreased growth at 30°C and a strong TS phenotype and both of these traits are partially rescued by overexpression of tRNA^Lys^_UUU_ alone and more significantly by combined overexpression of tRNA^Gln^_UUG_, tRNA^Glu^_UUC_ and tRNA^Lys^_UUU_ (Fig. 4B). Other than tRNA^Lys^_UUU_, however, single overexpression of tRNA^Glu^_UUC_ or tRNA^Gln^_UUG_ did not detectably suppress the TS phenotype of the *elp3uba4* double mutant (S3 Fig.), suggesting that among the three mcm^5^s^2^U carrying tRNAs, tRNA^Lys^_UUU_ is most dependent on this modification. Identical genetic interactions and suppression by excess tRNAs were also observed for *elp3* and *urm1* mutants (S4 Fig.).

To explore whether the negative genetic interaction of *elp3* and *urm1/uba4* and its suppression by excess levels of tRNA^Lys^_UUU_ applies to phenotypes other than TS, we analyzed growth responses to rapamycin, a TOR pathway inhibitor to which both, *elp3* and *urm1/uba4* mutants show enhanced sensitivity [22, 26]. The combination of *elp3* and *uba4* mutations led to a synergistic increase in rapamycin sensitivity in the *elp3uba4* double mutant, as clear growth impairment was observed on low concentrations of rapamycin which the single *elp3* and *uba4* mutants were able to tolerate (Fig. 4C). Resembling the suppressor effects on TS, overexpression of either tRNA^Lys^_UUU_ alone or in combination with tRNA^Gln^_UUG_ and tRNA^Glu^_UUC_ efficiently countered the negative genetic interaction of *elp3* and *uba4* and caused rapamycin resistance (Fig. 4C).

Next, we analyzed whether the observed suppressor effects of high copy tRNA^Lys^_UUU_ or the combination of tRNA^Gln^_UUG_, tRNA^Glu^_UUC_ and tRNA^Lys^_UUU_ on the growth phenotypes can be linked to a rescue of the observed effect on protein levels. Wild type cells and *elp3uba4* double mutants carrying empty vector or pK/pQKE plasmids were grown to exponential phase and identical cell numbers subjected to chemical lysis, followed by SDS-PAGE. When wild type and *elp3uba4* cells carrying the empty vector are compared, again a drastic loss of protein content was observed (Fig. 4D). Strikingly, the drop in cellular protein content is markedly countered by pQKE as well as pK plasmids (Fig. 4D). When Pfk1 levels were analyzed, we observed a partial rescue effect of pK and pQKE in the *elp3uba4* strain. However, both plasmids also detectably increased Pfk1 levels in the wild type background (Fig. 4D), suggesting that cellular pools of tRNA^Gln^_UUG_, tRNA^Glu^_UUC_ and tRNA^Lys^_UUU_ may be a limiting factor for expression of the highly abundant Pfk1 protein.

Together, these results demonstrate a strict correlation between growth phenotypes induced by tRNA modification defects and global cellular protein content. Partial or complete removal of wobble uridine modifications result in an increased severity of growth defects that goes along with decreased cellular protein content; thus, conditions that rescue growth defects, such as elevated tRNA levels also rescue the effect on cellular protein content, suggesting the decreased protein content causes the observed phenotypes. It remains to be determined whether effects on protein levels are a direct consequence of impaired translational elongation or whether the induction of cell death and additional cellular processes, such as enhanced protein turnover by protein quality control mechanisms or altered protein stabilities contribute to lower protein recovery from wobble uridine modification mutants.

## Discussion

Loss of Elongator function alters the modification states of 11 cytoplasmic tRNAs [3] by preventing the addition of ncm^5^ or mcm^5^ side chains at wobble uridines [2]. Of the affected tRNA species, 3 are normally destined to carry mcm^5^s^2^U_34_, which is generated by the joint action of the Elongator-controlled pathway and the Elongator independent tRNA thiolation pathway. Elongator and tRNA thiolation mutants display overlapping phenotypes, including TS and hypersensitivity to the TOR inhibitor rapamycin [19, 22, 24, 26]. The suppressor effect of elevated levels of tRNA^Lys^_UUU_ on the TS phenotype of *elp3* suggested malfunction of this tRNA to be mainly responsible for the growth defect and indicates that such malfunction can be compensated for by excess of the hypomodifed tRNA [24]. It was shown that loss of wobble uridine modifications does not affect abundance or aminoacylation of any of the 11 tRNA species targeted by Elongator- and/or thiolation pathways, indicating hypomodification-induced decoding rather than charging defects [3]. Genetic analysis further suggested that mcm^5^s^2^U primarily enhances decoding efficiency of the cognate, A-ending codons since under normal circumstances, there is no wobble decoding of the alternative G-ending codon by tRNA^Gln^mcm^5^s^2^_UUG_ or tRNA^Glu^mcm^5^s^2^_UUC_ [3]. The finding that combined loss of wobble uridine mcm^5^/ncm^5^ modification and thiolation has lethal effects in the *S*. *cerevisiae* W303-1B background, which can also be suppressed by excess of tRNA^Lys^_UUU_ suggested that mcm^5^ and s^2^ wobble uridine side chains are most critical for the function of this tRNA in decoding [17]. However, combined loss of wobble uridine mcm^5^ modification and thiolation is tolerated in *Caenorhabditis elegans* grown at 15°C [34] and also in fission yeast, an *elp3ctu1* (ortholog of *ncs6*) double deletion is viable [35], arguing against an absolute requirement of mcm^5^s^2^U for life in eukaryotes.

To reinvestigate this issue, we used a plasmid shuffle approach and analyzed synthetic interactions between Elongator and tRNA thiolation mutants in a non-W303 background. We show that in the S288C-derived reference strain BY4741, both modification pathways can be simultaneously inactivated at the expense of a synthetic growth defect and a TS-phenotype. The difference between the S288C- and W303-derived strains in tolerance to the complete removal of mcm^5^s^2^U could be linked to any of the 799 proteins predicted to exhibit sequence alterations in a W303 derived strain [29]. Simultaneous loss of both pathways in the BY4741 strain did not increase the ability of tRNA^Gln^_UUG_ to recognize the alternative G-ending (CAG) codon, suggesting that the mcm^5^s^2^ modification is not responsible for the inability of mcm^5^s^2^U carrying tRNAs to wobble decode G-ending codons, but may rather function to improve recognition of the cognate A-ending codon, as suggested previously [3]. While this work was in progress, another study reported the construction of viable *elp3uba4* double mutants in the prototrophic *S*. *cerevisiae* CEN.PK background, which resulted in the inactivation of metabolic cycling, a phenomenon that was not observed for either single mutant [36]. The existence of yet another non-W303 strain tolerating the entire removal of mcm^5^s^2^U [36] suggests that the lethal effects observed in the W303 strain [17] may represent an exception rather than the rule.

We also found additive or synergistic effects with respect to rapamycin-, caffeine-, SDS- and NaCl-sensitivity when combining *elp3* and *uba4* mutations (Figs. 4 and S5), suggesting the majority of single mutant phenotypes arise from a translational defect that becomes aggravated in the combined absence of mcm^5^ and s^2^ modifications. We checked the effects of mcm^5^s^2^U removal on total cellular protein levels in *S*. *cerevisiae* and observed a severe depletion of cellular protein content that was also exemplified for Cdc19 and Pfk1, usually two highly abundant proteins. RT-PCR assays excluded the possibility of a drastic transcriptional defect for *CDC19*, *PFK1* or *ACT1* genes in the *elp3uba4* double mutant, indicating the observed effects result from a disturbance of post-transcriptional events. Interestingly, many highly abundant proteins related to sugar- and carbohydrate metabolism, such as Tdh1, Tdh2, Adh1, Tpi1, Pgk1 and also Cdc19 were found in a genome wide SILAC approach to be significantly undertranslated in *S*. *cerevisiae* cells lacking *uba4* or *ncs2* [36, 37]. While we did not observe strong effects on total protein levels or Cdc19/Pfk1 in *uba4* alone, these data clearly support our conclusion that total protein levels are regulated by wobble uridine mcm^5^s^2^U modification, since partial removal (e.g. exchange of mcm^5^s^2^U to mcm^5^U) can already diminish highly abundant proteins [36]. Our data may suggest that this effect could be exacerbated upon complete removal of mcm^5^s^2^U. The removal of mcm^5^s^2^U could affect the efficiency of translational elongation directly, as both side chains (s^2^ and mcm^5^) were already shown to enhance the binding of tRNA^Lys^_UUU_ to the ribosomal A-site in vitro [27]. However, since SILAC experiments also revealed a clear induction of proteins involved in proteasomal degradation and protein folding/stability in *elp3/urm1* single mutants [27], increased protein degradation or decreased protein stability may also contribute to overall lowered protein detection in mcm^5^s^2^U lacking strains. Consistent with the interpretation that loss of wobble uridine modifications can cause protein stress, it was shown previously that *trm9* mutants lacking the methytransferase involved in mcm^5^ side chain formation activate certain heat shock proteins and markers of the unfolded protein response [38]. Additionally, *elp2*, *uba4*, *ncs2* and *ncs6* mutants were shown to accumulate ubiquitinated proteins and *urm1* as well as *trm9* mutants upregulate heat shock/protein stress transcripts *UBI4* and *SSA4*, possibly indicating protein stress in the absence of mcm^5^ or s^2^ uridine modification [38, 39, 40]. Also, the fact that the *elp3uba4* double mutant accumulates dead cells during exponential growth phase needs to be taken into consideration when interpreting results of reduced protein levels, in particular since a fraction of cells of this strain appears to undergo spontaneous cell lysis. Thus, effects of complete mcm^5^s^2^ removal on cellular protein levels are likely both, direct (affecting the translational elongation step) and indirect (inducing cell death and protein stress). Consistent with a key role of mcm^5^s^2^U in tRNA^Lys^_UUU_ for the maintenance of translational capacity, cellular protein levels were partially restored in the *elp3uba4* double mutant by overexpression of this tRNA. Thus, the severely reduced translational efficiency of tRNA^Lys^_UUU_ lacking mcm^5^s^2^ can be compensated by increasing its abundance. The easiest interpretation for this property is that absence of wobble uridine modification induces a binding defect of tRNA^Lys^_UUU_ to the ribosomal A-site (for which there is in vitro evidence [27]) and increasing the abundance of the affected tRNA can compensate for this defect. A related example, where overexpression of a weak decoder can improve its in vivo function is tRNA^Gln^_UUG_. As mentioned above, this tRNA is an inefficient CAG decoder due to the U/G wobble base pair in CAG:UUG codon anticodon interaction [3] and in complete absence of tRNA^Gln^_CUG_, cells die due to this decoding inefficiency. Upon overexpression of tRNA^Gln^_UUG_, however, cells can tolerate the absence of tRNA^Gln^_CUG_ [3], indicating that elevated levels of the inefficient CAG decoder can compensate this defect. The finding that single overexpression of tRNA^Lys^_UUU_ but not tRNA^Gln^_UUG_ or tRNA^Glu^_UUC_ can detectably suppress growth defects of *elp3uba4* mutants (Figs. 4B and S3) indicates that upon removal of mcm^5^s^2^U, tRNA^Lys^_UUU_ is the most affected one among the three tRNAs carrying this modification. However, since growth defects are most efficiently rescued by combined overexpression of all three tRNAs normally carrying mcm^5^s^2^ (Fig. 4B), it is also evident that all of them are affected to some extent by loss of the modification.

Since there is a close correlation between the severity of growth phenotypes and cellular protein content and both, growth defects and diminished protein levels can be rescued by elevated copy numbers of tRNA^Lys^_UUU_, we conclude that the reduced protein levels in absence of mcm^5^s^2^U are the cause of the observed growth phenotypes. A recent study in *S*. *cerevisiae* determined that individual loss of wobble uridine mcm^5^/ncm^5^ modification or thiolation affects translational efficiency depending on transcript codon usage, where translation of transcripts enriched for AAA-, CAA-, and GAA-codons was specifically affected [27]. Another genome wide study indicated these effects on AAA, CAA and GAA decoding would be too small to affect protein output [28]. Our findings of reduced protein levels in *elp3uba4* cells and the suppression of this effect by excess of tRNA^Lys^_UUU_ may support a role of AAA codon usage in regulating the translational capacity. However, a severe malfunction of any tRNA that cannot be replaced by an alternative isodecoder would result in a global, rather than codon-specific translational defect. The strong growth and protein defects in the *elp3uba4* double mutant and their suppression by tRNA^Lys^_UUU_ indicate that in the absence of mcm^5^s^2^U, this tRNA becomes defective enough to affect protein levels in a manner largely independent of AAA codon frequency. We conclude that individual loss of mcm^5^ and s^2^ modifications in tRNA cause rather subtle translational defects that may affect the proteome differentially based on codon frequency, whereas the complete loss of mcm^5^s^2^ results in a more global defect in protein homeostasis that may be caused by defective translational elongation and/or the induction of cellular stress pathways. In support of the latter option, individual loss of mcm^5^ or s^2^ wobble uridine modifications was shown to activate the general aminoacid control pathway, a consequence of which is the inhibition of global translation, and interfere with TOR pathway signaling [28, 41]. Since tRNA thiolation and mcm^5^ side chain formation are independent pathways and were shown to respond to different environmental or endogenous stimuli such as sulfur availability, cell cycle progression and temperature stress [36, 40, 42, 43, 44], future work will need to decipher how cross talk between both pathways manages to produce different outputs on translational efficiency, ranging from moderate, codon specific to more drastic global effects.

## Materials and Methods

### Strains, general methods and constructs

Strains used in this study are listed in S1 Table. Standard methods were used for growth and maintenance [45]. To select for plasmids, synthetic complete (SC) medium lacking the appropriate nutrients was employed. To select cells having lost *URA3* containing plasmids, 5-fluoro-orotate containing (1g/L) SC medium was used. Transformation of *S*. *cerevisiae* was performed by the PEG/lithium acetate method [46]. Genomic deletions were generated by PCR using template plasmids pYDp-H [47] or pUG73 [48] and oligonucleotides targeting *UBA4*, *URM1* or *tQ(CUG)M/SUP70* (S2 Table). Replacements were verified by PCR using primers located outside the target genes (S2 Table). For overexpression of tRNA, previously described constructs pDJ83/pK (tRNA^Lys^_UUU_), YEpQ (tRNA^Gln^_UUG_), pSZ16 (tRNA^Glu^_UUC_) and pQKE (tRNA^Lys^_UUU_, tRNA^Glu^_UUC_, tRNA^Gln^_UUG_) were utilized [21, 23, 25]. Generation of genomic deletions of *tQ(CUG)M/SUP70* was facilitated by transformation with pAK01 (*URA3*, *CEN*) carrying the *tQ(CUG)M/SUP70* gene [49]. Wild type *ELP3* was amplified from chromosomal DNA by PCR using oligonucleotides FF21 and FF22 and cloned into YCplac33, yielding pFF8. For growth phenotypes, cultures were adjusted to identical OD_600nm_ readings and serial dilutions corresponding to OD_600nm_ 0.15, 0.015, 0.0015 and 0.00015 spotted onto YPD plates with or without addition of various concentrations of rapamycin.

### Cell viability measurements

Yeast strains were grown to early exponential phase (OD_600nm_ ∼0.5) in liquid YPD harvested by centrifugation and resuspended in sterile water to OD_600nm_ = 1. Aliquots were removed for total cell counting using a Neubauer chamber and serially diluted and plated on YPD. Colonies were counted after 3 days at 30°C and values obtained used to calculate the viable cell titer. Data presented are the mean of four independent experiments with standard deviation. Dead cell staining involved the incubation of a culture aliquot with one volume of methylene blue solution (0.1 g/L methylene blue, 20 g/L sodium citrate dihydrate) for 5 min at room temperature and subsequent microscopic observation using phase contrast and bright field optics.

### Protein isolation and Western blotting

Preparation of protein extracts was done using either mechanical disruption with glass beads [50] or chemical lysis [51]. Protein extracts obtained via glass bead lysis were quantified using Bradford assay [52] to ensure equal loading of gels. For chemical lysis, cultures were first adjusted to identical OD_600nm_ values and 20 μl removed for serial dilution and spot assay analysis on YPD (to control for equal densities of viable cells prior to lysis). The remaining suspension was lysed using NaOH treatment and boiling in SDS sample buffer as described [51]. Equal volumes of these lysates were loaded on gels for Western analysis. Transfer and detection was done as described previously [19] and involved anti-Pfk1 (kindly provided by Dr. J. Heinisch) and anti-Cdc19 (kindly provided by Dr. J. Thorner) antibodies.

### tRNA isolation and modification status by HPLC-DAD-MS/MS analysis

Total tRNA was isolated from yeast cultures as described previously [53]. Prior to LC-MS/MS analysis, 5 μg of each tRNA sample (*wt*, *elp3*, *uba4*, *elp3uba4*) were digested into nucleosides according to the following protocol: samples were incubated in presence of 1/10 volume of 10x nuclease P1 buffer (0.2 M NH_4_OAc pH 5.0, ZnCl_2_ 0.2 mM), 0.3 U nuclease P1 (Sigma Aldrich, Munich, Germany) and 0.1 U snake venom phosphodiesterase (Worthington, Lakewood, USA) at 37°C for 2 h. Next, 1/10 volume of 10x fast alkaline phosphatase buffer (Fermentas, St. Leon-Roth, Germany) and 1 U fast alkaline phosphatase (Fermentas, St. Leon-Roth, Germany) were added, and samples were incubated for additional 60 min at 37°C. The digested tRNA samples were analyzed on an Agilent 1260 HPLC series equipped with a diode array detector (DAD) and a triple quadrupol mass spectrometer (Agilent 6460). A Synergy Fusion RP column (4 μm particle size, 80 Å pore size, 250 mm length, 2 mm inner diameter) from Phenomenex (Aschaffenburg, Germany) was used at 35°C column temperature. The solvents consisted of 5 mM ammonium acetate buffer adjusted to pH 5.3 using acetic acid (solvent A) and pure acetonitrile (solvent B). The elution was performed at a flow rate of 0.35 ml/min using a linear gradient from 0% to 8% solvent B at 10 min, 40% solvent B at 20 min and 0% solvent B at 23 min. For additional 7 min, the column was rinsed with 100% solvent A to restore the initial conditions. Prior to entering the mass spectrometer, the effluent from the column was measured photometrically at 254 nm by the DAD for the detection of the 4 canonical nucleosides. The triple quadruple mass spectrometer, equipped with an electrospray ion source (Agilent Jet Stream), was run at the following ESI parameters: gas (N_2_) temperature 350°C, gas (N_2_) flow 8 L/min, nebulizer pressure 50 psi, sheath gas (N_2_) temperature 350°C, sheath gas (N_2_) flow 12 L/min and capillary voltage 3000 V. The MS was operated in the positive ion mode using Agilent MassHunter software and modified nucleosides were monitored by multiple reaction monitoring (dynamic MRM mode). For identification of s^2^U and retention time determination, comparison to a synthetic standard (Berry & Associates, Dexter, USA) was used. Identification of ncm^5^U, mcm^5^U and mcm^5^s^2^U peaks were performed as described previously [54]. All mass transitions and retention times used for identification of the modified nucleosides can be found in S3 Table. Peak areas were determined employing Agilent MassHunter Qualitative Analysis Software. In the case of the major nucleosides, peak areas were extracted from the recorded UV chromatograms in order to avoid saturation of the mass signals. For intersample comparability of the detected modifications, the peak areas of the modified nucleosides were normalized to the UV peak area of uridine.

### RT-PCR

Total yeast RNA was isolated using TRIzol reagent (ambion) as recommended by the manufacturer and subsequently treated with RNase free DNase (Thermo Scientific). RNA preparations were checked for remaining DNA by PCR amplification. For reverse transcription, the RevertAid first strand cDNA synthesis kit (Thermo scientific) was used with 1–4 μg total RNA and random hexamer primers in a reaction volume of 20 μl. 1/20 of the cDNA was subsequently analyzed by PCR using oligonucleotides act1fw/act1rv, cdc19fw/cdc19rv and pfk1fw/pfk1rv (S2 Table).

### Fluorescence microscopy

Cells were fixed by adding 3.7% formaldehyde directly to the medium and incubated for 10 min at room temperature. Subsequently, cells were resuspended in water containing 1 μg ml^-1^ 4,6 diamidino-2-phenylindole (DAPI, Sigma, Germany). Following washing with water, cells were analyzed using an Olympus BX53 microscope with appropriate filters. Cell length was measured using the CellSens 1.6 software package (Olympus).

## Supporting Information

S1 Fig Microscopy of WT and *elp3uba4* mutants. Cells were fixed and stained with DAPI. l indicates average cell length with standard deviation in parentheses. n indicates the number of cells analyzed for cell length determination. White triangles indicate sporadically occurring elongated cell types in the *elp3uba4* double mutant.
(EPS)

S2 Fig Total and viable cell titers in OD_600nm_-adjusted cultures. Exponential cultures of wild type (WT) and *elp3uba4* were adjusted to OD600nm = 1 and total and viable cell titers determined. Values presented are the mean of four independent experiments. (A) Total cell counts and deviation in %. (B) Viable cell counts and deviation in %. (C) Methylene blue staining of inviable cells. Left row: phase contrast (phase), right row: brightfield (methylene blue).
(EPS)

S3 Fig Elevated levels of tRNA^Gln^_UUG_ and tRNA^Glu^_UUC_ alone fail to suppress thermosensitivity of the *elp3uba4* double mutant. Serial dilution and spot assay was done with WT and *elp3uba4* strains overexpressing no tRNA (-), tRNA^Gln^_UUG_ or tRNA^Glu^_UUC_. Replicas were incubated at 30°C or 37°C and photographed after 32h and again after 46h of incubation.
(EPS)

S4 Fig tRNA overexpression rescues temperature sensitivity of the *elp3urm1* double mutant. Serial dilution and spot assay with indicated strains carrying no plasmid, empty vector (vector), or vectors overexpressing tRNA^Lys^_UUU_ alone (pK) or together with tRNA^Gln^_UUG_ and tRNA^Glu^_UUC_ (pQKE). Replicas were incubated at 30°C or 37°C and photographed after 24h and again after 36h of incubation.
(EPS)

S5 Fig Negative genetic interaction of *elp3* and *uba4* with respect to exogenous stresses. Serial dilution and spot assay with indicated strains on plates containing the indicated additions. Plates were incubated for 42h at 30°C and photographed.
(EPS)

S1 Table Strains used in this study. (DOCX)

S2 Table Oligonucleotides used in this study. (DOCX)

S3 Table QQQ parameters of the dynamic MRM method. (DOCX)
