# Supplementary material for: Loss of Anticodon Wobble Uridine Modifications Affects tRNALys Function and Protein Levels in Saccharomyces cerevisiae
Source: PLoS One. 2015 Mar 6;10(3):e0119261. doi: 10.1371/journal.pone.0119261 (PMC4352028; doi:10.1371/journal.pone.0119261)
Supplement: S1 Table — (DOCX) [file pone.0119261.s006.docx]

S1 Table

| Strain | Genotype | Reference/source |
| --- | --- | --- |
| *S. cerevisiae* BY4741 | MATa, *his3Δ, leu2Δ, met15Δ, ura3Δ* | Euroscarf, Frankfurt |
| *S. cerevisiae* elp3 | BY4741 *elp3ΔKanMX4* | Euroscarf, Frankfurt |
| *S. cerevisiae* urm1 | BY4741 *urm1ΔKanMX4* | Euroscarf, Frankfurt |
| *S. cerevisiae* uba4 | BY4741 *uba4ΔKanMX4* | Euroscarf, Frankfurt |
| *S. cerevisiae* trm9 | BY4741 *trm9ΔKanMX4* | Euroscarf, Frankfurt |
| *S. cerevisiae* elp3 uba4 | BY4741 *elp3ΔKanMX4 uba4ΔHIS3* | this work |
| *S. cerevisiae* elp3 urm1 | BY4741 *elp3ΔKanMX4 urm1ΔHIS3* | this work |
| *S. cerevisiae* sup70 | BY4741 pAK01 *sup70*Δ*KlLEU2* | this work |
| *S. cerevisiae* elp3 uba4 sup70 | BY4741 *elp3ΔKanMX4 uba4ΔHIS3* pAK01 *sup70*Δ*KlLEU2* | this work |
