# Supplementary material for: Loss of Anticodon Wobble Uridine Modifications Affects tRNALys Function and Protein Levels in Saccharomyces cerevisiae
Source: PLoS One. 2015 Mar 6;10(3):e0119261. doi: 10.1371/journal.pone.0119261 (PMC4352028; doi:10.1371/journal.pone.0119261)
Supplement: S2 Table — (DOCX) [file pone.0119261.s007.docx]

S2 Table

| Oligonucleotide | Sequence | Target |
| --- | --- | --- |
| koUBA4fw | GTAAATAGAAGTCAATAACAATGAATGACTACCATCT  CGAGGATACCACGCGACGGCCAGTGAATTCCCGG | YDp-H/*UBA4* |
| koUBA4rv | ATGGTTTGATCAATATCGTCTATGTATTTGAAGTATCC  TCCTCTCACGTCAGCTTGGCTGCAGGTCGACGG | YDp-H/*UBA4* |
| uba4fw | CTTCTATTCGTAAGGAGCAAGACGC | *UBA4* |
| uba4rv | GCAGGATGCAGCCTGAATAAATTGC | *UBA4* |
| koURM1fw | AAAACGAGATAGGTTAATAGCAAAATCGGGATGGTAAACGTGAAAGTGGACGACGGCCAGTGAATTCCCGG | YDp-H/*URM1* |
| koURM1rv | TTAACCACCATGTAATGTTGAAGTAAAAGAGATGATGTCACCGTCTTCCAAGCTTGGCTGCAGGTCGACGG | YDp-H/*URM1* |
| urm1fw | CACATACCGGATTATGTTCCTTCCC | *URM1* |
| urm1rv | ACTGGAATGGTAGAGGTCTTTTGGG | *URM1* |
| sup70koF | AGGTTCCATAAAACCGGAAGTTTTAGTGTACACTAACAACAGAAGAAAAACAGCTGAAGCTTCGTACGC | pUG73 |
| sup70koR | AAAAAAAAAAATGATGGTTTAAATTTCGTAAAATACGAAAAATGAAGGGAGCATGCATAGGCCACTAGTGGATCTG | pUG73 |
| sup70fw | ATGAGGAGAGCTTCTACTA | *SUP70* |
| sup70rv | CATAATGATGAGCACTTGTC | *SUP70* |
| FF21 | CGATAAGACAGTGAGAGAAGG | *ELP3* |
| FF22 | AACACATGCAGCAGTTACTCC | *ELP3* |
| act1fw | CTTCCGGTAGAACTACTGGT | *ACT1* |
| act1rv | CCTTACGGACATCGACATCA | *ACT1* |
| cdc19fw | CTTGGGTGAACAAGGTAAGG | *CDC19* |
| cdc19rv | AGGTTGGCTTTGGAGTACAG | *CDC19* |
| pfk1fw | TGGTACTGCTCGTTCTATGG | *PFK1* |
| pfk1rv | GGCACACTTCCTTCAATTCG | *PFK1* |
