# Supplementary material for: Loss of Anticodon Wobble Uridine Modifications Affects tRNALys Function and Protein Levels in Saccharomyces cerevisiae
Source: PLoS One. 2015 Mar 6;10(3):e0119261. doi: 10.1371/journal.pone.0119261 (PMC4352028; doi:10.1371/journal.pone.0119261)
Supplement: S3 Table — (DOCX) [file pone.0119261.s008.docx]

**S3 Table**

| **Modified nucleoside** | **Precursor ion**  **m/z** | **Product ion**  **m/z** | **Fragmentor voltage [V]** | **Collision energy [eV]** | **Cell accelerator voltage [V]** | **Retention time [min]** | **∆rt**  **[ min]** |
| --- | --- | --- | --- | --- | --- | --- | --- |
| mcm^5^s^2^U | 333 | 201 | 66 | 5 | 2 | 16.3 | 2 |
| mcm^5^U | 317 | 185 | 66 | 5 | 2 | 12.7 | 2 |
| ncm^5^U | 302 | 170 | 66 | 5 | 2 | 6.0 | 2 |
| s^2^U | 261 | 129 | 66 | 5 | 2 | 11.3 | 2 |
